# MOF-Mediated Synthesis of CuO/CeO_2_ Composite Nanoparticles: Characterization and Estimation of the Cellular Toxicity against Breast Cancer Cell Line (MCF-7)

**DOI:** 10.3390/jfb12040053

**Published:** 2021-09-28

**Authors:** Mohammad Javad Farhangi, Ali Es-haghi, Mohammad Ehsan Taghavizadeh Yazdi, Abbas Rahdar, Francesco Baino

**Affiliations:** 1 Department of Biology, Mashhad Branch, Islamic Azad University, Mashhad 91871-47578, Iran; Farhangi.mohamadjavad123@gmail.com (M.J.F.); ashaghi@gmail.com (A.E.-h.); 2 Applied Biomedical Research Center, Mashhad University of Medical Sciences, Mashhad 91388-13944, Iran; 3 Department of Physics, University of Zabol, Zabol 98613-35856, Iran; 4 Institute of Materials Physics and Engineering, Applied Science and Technology Department, Politecnico di Torino, Corso Duca Degli Abruzzi 24, 10129 Torino, Italy

**Keywords:** nanomaterials, copper oxide, cerium oxide, metal–organic frameworks, anticancer, MCF-7

## Abstract

A copper oxide/cerium oxide nanocomposite (CuO/CeO_2_, NC) was synthesized via a novel method using a metal–organic framework as a precursor. This nanomaterial was characterized by Fourier transform infrared spectroscopy (FTIR), powder X-ray diffraction (PXRD), field emission scanning electron microscopy (FESEM), transmission electron microscopy (TEM), dynamic light scattering size analysis (DLS), and zeta potential. The PXRD showed the successful synthesis of the CuO/CeO_2_ NC, in which the 2theta values of 35.55° (d = 2.52 Å, 100%) and 38.73° (d = 2.32 Å, 96%) revealed the existence of copper (II) oxide. FTIR analysis showed the CeO_2_, hydroxyl groups, absorbed water, and some residual peaks. The solid phase analysis by FESEM and TEM images showed mean particle sizes of 49.18 ± 24.50 nm and 30.58 ± 26.40 nm, respectively, which were comparable with crystallite size (38.4 nm) obtained from PXRD, but it appears the CuO/CeO_2_ NC was not evenly distributed and in some areas, showed it was highly agglomerated. The hydrodynamic size (750.5 nm) also showed the agglomeration of the CuO/CeO_2_ NCs in the solution, which had a negatively charged surface. The CuO/CeO_2_ NCs showed anti-proliferative activity against human breast cancer cell line (MCF-7) in a dose- and time-dependence way, while affecting normal cells less significantly.

## 1. Introduction

The increased resistance of cancers to conventional treatments has become problematic [1,2,3,4]. The resistance of cancer cells to chemical drugs leads to a decrease in the response level of these cells to the drug and consequently the failure of the treatment. Therefore, the development of more effective drugs with few side effects and limitations is very important [5,6,7,8]. Nanotechnology can provide physicians with new strategies for directly targeting cancer cells and increasing drug efficacy [9,10]. Biomaterials are used in various cases such as drug delivery as well as imaging applications and have good potential for cancer diagnosis and treatment [11,12,13,14]. Pharmaceuticals can bind to nanoparticles (NPs) and the assemblies are specifically absorbed by the cancer cells by passive targeting. With this method, healthy cells are not exposed to pharmaceuticals and the side effects of the drug are reduced [15,16,17].

In this context, cerium oxide (ceria) nanoparticles are among the most promising. Recent studies have shown that this nanomaterial is cytotoxicity against cancer cells, so further studies are important for determining side effects and its use in the treatment of cancers [18,19,20,21]. The popularity of cerium oxide (CeO_2_) is increasing in biomedical applications, which comes from the intrinsic properties of the ceria such as its oxidation–reduction behavior due to the surface oxygen vacancies and reversible valence state changes from Ce (III) to Ce (IV) [22,23]. The reduction of the particle size to nano-scale dimensions has a tremendous effect on its catalytic behavior. The expansion of synthesis methods that provide control over final morphology and size gives a new ability to this material, especially for medical-related applications [24,25,26]. Cerium oxide nanoparticles are widely used in various fields such as catalysis, gas sensors, fuel cells, hydrogen storage materials, optical devices, ultraviolet absorbents, polishing material, and many fields of biomedical science [27,28,29,30,31,32,33,34,35,36]. It has been proved that ceria nanoparticles are effective against oxidative stress and have an antioxidant role. Cerium oxide nanoparticles (nanoceria) are able to mimic the activity of superoxide dismutase and catalase due to changes in oxidation state [37,38]; therefore, these nanoparticles can be used as scavengers for reactive oxygen species (ROS) and inhibitors of the invasion and sensitization of cells to radiotherapy and chemotherapy [39,40,41]. Cerium oxide nanoparticles have cytotoxic effects against cancer cells and can induce apoptosis in them. One mechanism is cytochrome C release and caspase-3 and -9 activations. In fact, nanoceria increases apoptosis in cancer cells by the onset of mitochondrial cell death without chemical changes by targeting mitochondria [42]. In general, induction of programmed cell death or apoptosis is one of the most attractive approaches in cancer treatment. The cell death pathway can involve activation of pro-apoptotic events in the cell, which begin with the permeability of the mitochondrial membrane by Bax and Bak proteins, releasing cytochrome C from it and finally activating caspase-9 and then caspase-3 [43].

It was shown that the use of copper oxide nanoparticles, too, reduces the secretion of superoxide dismutase and catalase enzymes [44]. As oxidative stress was increased, the toxicity of nanoparticles was increased through ROS production. Therefore, copper oxide can be used as a dopant to adjust the antioxidant attributes, ROS production and catalytic activity of cerium oxide and make it more toxic against cancerous cells or bacteria. Mixed metal oxide nanocomposites (NCs) show characteristics of two metal oxides concurrently and may give an outstanding catalytic performance to the composite against cancer. Due to the importance of size, shape and morphology on the medical applications, the NCs produced in this study were analyzed comprehensively by investigating their physicochemical properties; cell toxicity tests with cancer cells were reported as well.

## 2. Materials and Methods

### 2.1. Reagents

All the chemicals and materials including terephthalic acid, dimethylformamide (DMF), ammonium cerium (IV) nitrate, and copper (II) nitrate trihydrate were procured from Sigma and Merck chemical groups unless otherwise stated.

### 2.2. Synthesis of the CuO/CeO_2_ NCs Nanocomposites (NCs)

The precursor was prepared according to previous reports and used without any further purification [41]; the final CuO/CeO_2_ NCs were synthesized thermally for the first time. In brief, 1000 mg of terephthalic acid was sonicated in 34 mL of DMF until it was dissolved. Then, 3302 mg of ammonium cerium (IV) nitrate and 70 mg of copper (II) nitrate trihydrate were dissolved in DMF and added to the above mixture, which was stirred at 100 °C for 15 min. The formed precipitate was centrifuged, washed with DMF several times and dried in an oven at 80 °C. Then, 2 g of the prepared precursor was heated at 500 °C for 4 h. The obtained nanopowder underwent characterization and was used for anticancer experiments.

### 2.3. Characterization of Nanoparticles

The used techniques were transmission electron microscopy (TEM, ZEISS LEO 912 AB, Oberkochen, Germany) and field emission scanning electron microscopy (FESEM, TESCAN, MIRA 3, Brno, Czech Republic), which were used for the analyses of size and morphology (using an ultrasonic probe 20 kHz and 400 W power for 30 min to prepare the sample for analyses), Fourier transform infrared spectroscopy (FTIR, Shimadzu 8400, Kyoto, Japan) for determination of functional groups by using KBr pellet in the range of wave number (4000–400 cm^−1^), powder X-ray diffraction (PXRD, D8 ADVANCE-BRUKER) to determine the structure by using a Cu Kα radiation (λ = 1.5406 Å) within 10–70° (2θ) range, dynamic light scattering size analysis (DLS, Particle Size Analyzer, Vasco3, Cordouan Technologies, Pessac, France) for the measurement of hydrodynamic sizes using a 100 mg/L concentration of the nanoparticles, and Zeta-potential (Zeta Compact, CAD) for the assessment of surface charges.

### 2.4. In Vitro Cellular Tests

Human breast carcinoma cell line (MCF-7) was selected as a suitable in vitro model of solid tumors. MCF-7 cells were achieved from Pasteur Institute, Iran and was grown in Dulbecco’s Modified Eagle Medium (DMEM) including 10 percentages of fetal bovine serum (FPS), 1 percentage of antibiotic (Pen/Str), and it was kept at 37 °C, 5% CO_2_, and 95% humidity. The cells were treated with different concentrations (0.031, 0.062, 0.125, 0.250, 0.500, and 1.000 μg/mL) of synthesized CuO/CeO_2_ NCs at three time points (24, 48, and 72 h). After incubation, the cell state was observed using a microscope, and later, 20 μL MTT substance was added and incubated. A 96-cell plate reader was used to estimate the absorbance at 570 nanometer and the cell vitality percentage amount was calculated. Mouse embryonic fibroblasts (NIH-3T3) cell lines were used as normal cells and cultured under the same conditions described above.

### 2.5. Statistical Analysis

Data were analyzed using GraphPad Prism 6.0 (GraphPad software, Inc., San Diego, CA, USA). Data were presented as mean ± standard deviation of at least three independent experiments. Student’s *t*-test was performed for comparison between groups. *p* < 0.05 was considered statistically significant.

## 3. Results and Discussion

### 3.1. Fourier-Transform Infrared Spectroscopy (FTIR)

**Figure 1:**
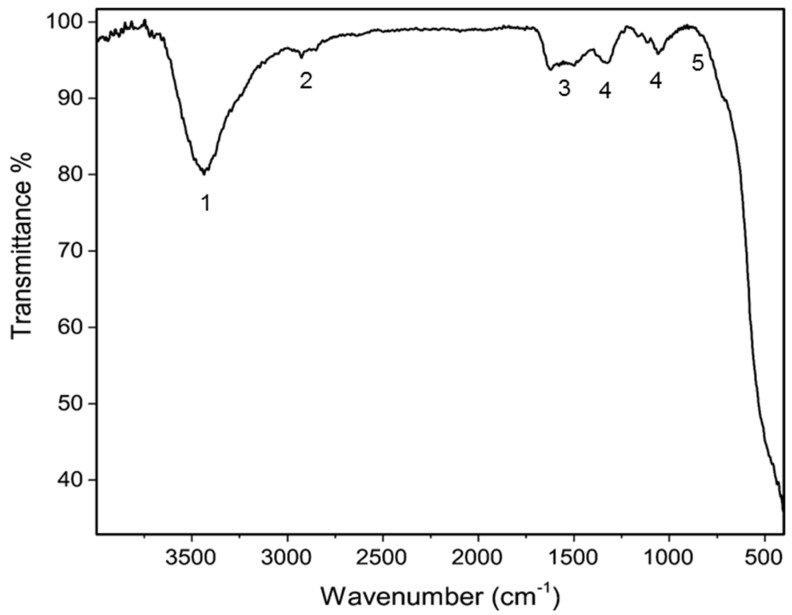
The FTIR spectrum of the CuO/CeO_2_ NCs (1, absorbed H_2_O or O–H stretching; 2, C–H stretching; 3, O–H stretching; 4, Ce–O–Ce vibration; 5, Ce–O/Cu–O vibration).

The FTIR spectrum of the CuO/CeO_2_ NCs was recorded from 400 to 4000 cm^−1^ to analyze the functional groups (Figure 1). The observed band at 3437 cm^−1^ was associated with absorbed water or hydroxyl groups on the surface. The appeared bands at 2800–3000 cm^−1^ is related to the presence of methylene groups from the residual organic groups after calcination [45]. The absorption band at 1621 cm^−1^ corresponded to the bending vibration of the hydroxyl groups or the absorbed H_2_O [46,47]. The peaks area under 800 cm^−1^ was related to Ce-O or Cu-O bond vibrations [48]. The bands at 1319 cm^−1^ and 1059 cm^−1^ could show the vibrational modes of Ce-O-Ce [49].

### 3.2. Powder X-ray Diffraction (PXRD)

**Figure 2:**
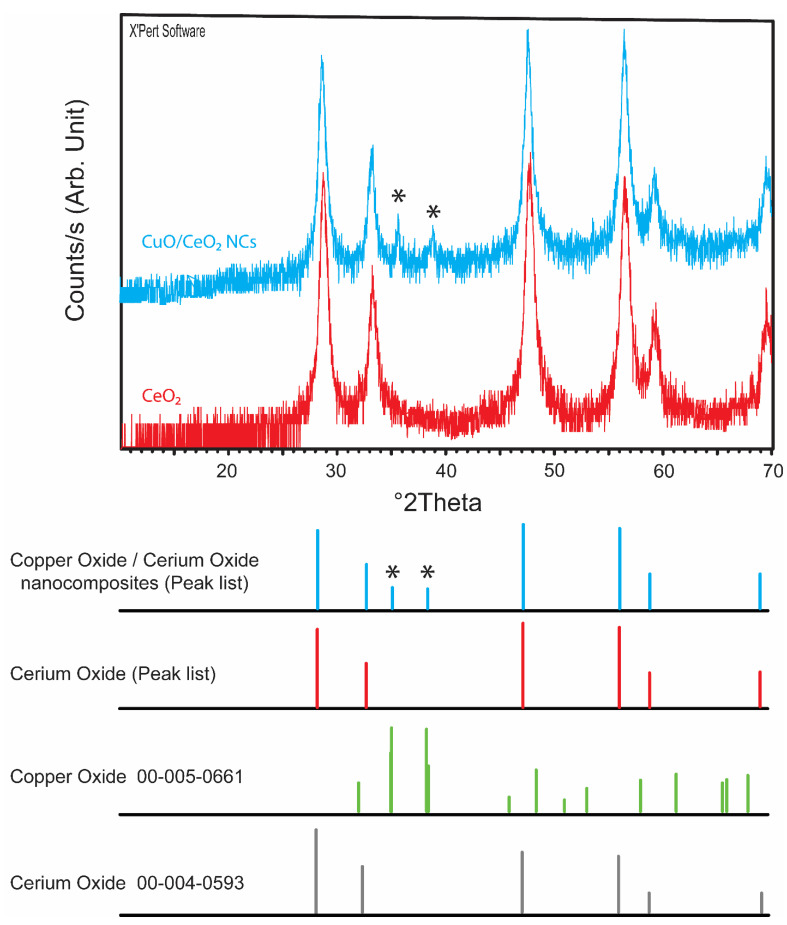
PXRD analyses of the CeO_2_ NPs and CuO/CeO_2_ NCs. * highlight the peaks interpreted according to the bottom part of the figure.

The PXRD analysis was performed to investigate the structural changes after copper doping (Figure 2). CeO_2_ and CuO were compatible with reference codes of 00-004-0593 and 00-005-0661, respectively. It appears that the crystal system of CeO_2_ NPs was cubic and associated with 2theta values of 28.55° (d = 3.12 Å, 100%), 33.08° (d = 2.71 Å, 29%), 47.49° (d = 1.91 Å, 51%), 56.33° (d = 1.63 Å, 44%), 59.10° (d = 1.56 Å, 5%) and 69.41° (d = 1.35 Å, 5%) and hkl values of (111), (200), (220), (311), (222), and (400) respectively. The experimental values of CeO_2_ NPs were 28.63° (d = 3.12 Å, 99.5%), 33.15° (d = 2.70 Å, 34%), 47.51° (d = 1.91 Å, 100%), 56.44° (d = 1.63 Å, 95%), 59.04° (d = 1.56 Å, 18%), and 69.53° (d = 1.35 Å, 16%), and the values of CuO/CeO_2_ NCs were 28.58° (d = 3.12 Å, 86%), 33.26° (d = 2.69 Å, 28%), 35.61° (d = 2.52 Å, 6%), 38.85° (d = 2.32 Å, 5.5%), 47.60° (d = 1.91 Å, 100%), 56.42° (d = 1.63 Å, 91%), 59.17° (d = 1.56 Å, 17%), and 69.55° (d = 1.35 Å, 7%). The results showed the phase purity of CeO_2_ NPs. The 2theta values of 35.61° and 38.85° in CuO/CeO_2_ NCs were ascribed to the copper oxide with a monocilinic structure and reference code of 00-005-0661, which were associated to the 2theta values of 35.55° (d = 2.52 Å, 100%) and 38.73° (d = 2.32 Å, 96%), and hkl values of (−111) and (111), respectively. Hence, it can be concluded that CuO/CeO_2_ NCs were successfully synthesized after calcination. No additional peaks were observed other than those of CuO and CeO_2_, which indicated the phase purity of the prepared NCs. The crystallite sizes obtained by the Scherer’s equation using the most intense peaks were 32.9 nm and 38.4 nm for CeO_2_ NPs and CuO/CeO_2_ NCs, respectively. It appears that the crystallite size was increased after copper doping into the CeO_2_ nanostructures. 

### 3.3. Field Emission Scanning Electron Microscopy (FESEM)

**Figure 3:**
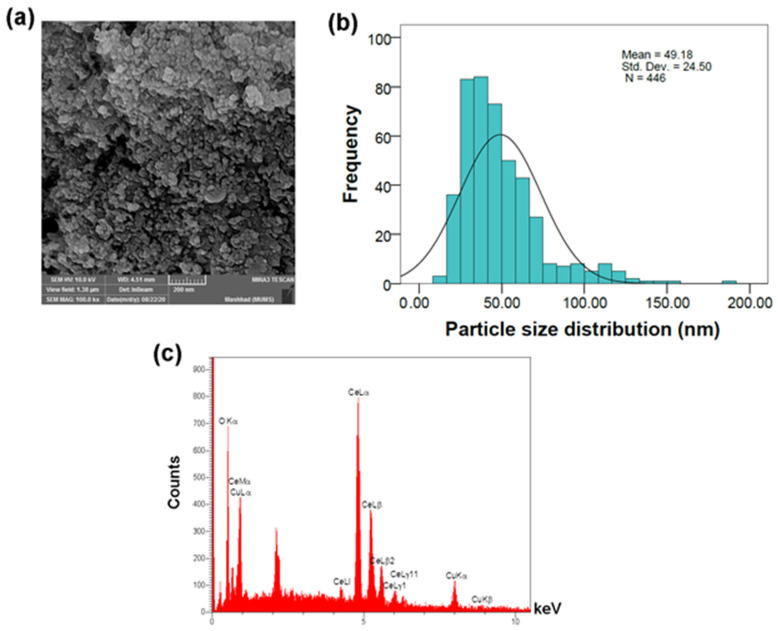
(**a**) FESEM images, (**b**) particle size distribution and (**c**) EDX analysis of the CuO/CeO_2_ NCs.

The FESEM images were used to analyze the morphology of the NCs (Figure 3). It appears that the particles have a spherical morphology and the mean particle size of the CuO/CeO_2_ NCs was 49.18 ± 24.50 nm. The particle sizes were determined by ImageJ software (bundled with Java 1.8.0_172) and particle size analyses were performed using IBM SPSS statistics 22. The maximum, minimum, and overall ranges were 189.73 nm, 12.55 nm and 177.18 nm, respectively. The median (44.15 nm) was less than the mean size of the NCs and greater than the mode (27.46 nm), which indicated a positively skewed frequency distribution and the skewness was 1.78. The difference between crystallite size (38.4 nm) and the mean particle size obtained from FESEM images (49.18 nm) indicated very low aggregation. The EDX analysis also showed the elemental composition of the copper and cerium in NC. The O Kα, Ce Lα, and Cu Kα were the most intense peaks related to oxygen, cerium and copper, respectively, which appeared at 0.53, 4.82, and 8.01 keV. 

### 3.4. Transmission Electron Microscopy (TEM)

**Figure 4:**
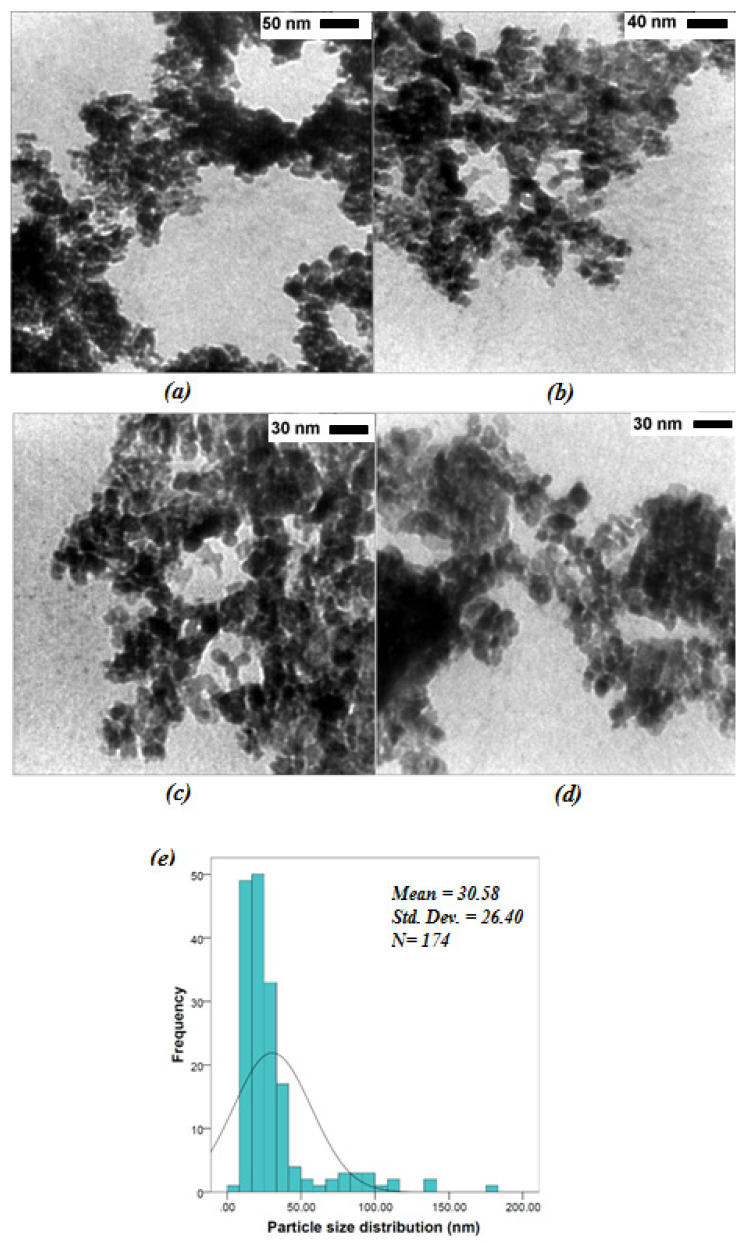
TEM images (**a**–**d**) and particle size distribution (**e**) of the CuO/CeO_2_ NCs.

The TEM images (Figure 4) showed that CuO/CeO_2_ NCs were spherical and the particle diameter was 30.58 ± 26.40 nm. It appears that the mode, median, and mean showed the following order: mode < median < mean (13 < 22.98 < 30.58 nm), which indicated a positively skewed frequency distribution and skewness was 2.76. The minimum and maximum were calculated to be 8.22 nm and 170.73 nm, respectively. Although the difference between crystallite size (38.4 nm) and the grain sizes obtained from TEM images (30.6 nm) suggests low agglomeration, the presence of large particle (~178 nm) indicated that the CuO/CeO_2_ NCs were not evenly distributed and the sample was highly agglomerated at some points. Therefore, the CuO/CeO_2_ NCs were well-sonicated before undergoing biological in vitro tests.

### 3.5. Dynamic Light Scattering (DLS) and Zeta Potential

**Figure 5:**
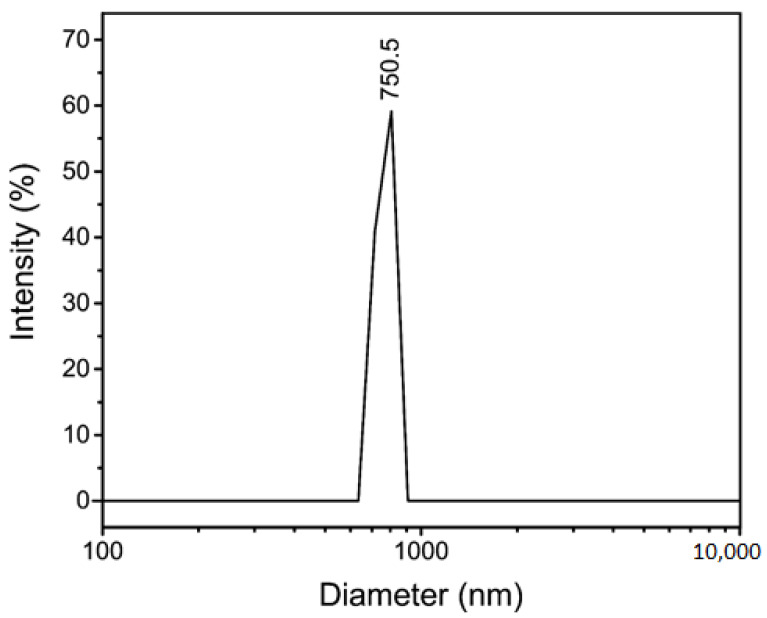
Dynamic light scattering (DLS) of CuO/CeO_2_ NCs.

The DLS analysis (Figure 5) showed that CuO/CeO_2_ NCs were highly agglomerated. Compared to the solid-state size, the hydrodynamic size (750.5 nm) was more than 15- and 24-times greater than the mean sizes obtained from FESEM and TEM images, respectively. The measurement of the hydrodynamic particle sizes presented the sizes of the largest particles in the solution. The weak interactions of the solvent such as hydrogen bonds with the surface of the CuO/CeO_2_ NCs led to the formation of layers of waters, aggregation of smaller particles or maybe other ionic components around the NCs. Therefore, the increased hydrodynamic size is reasonable. The Zeta potential was also assessed to be −20.0 mV, which was due to the presence of the hydroxyl or maybe the carboxyl groups on the surface of the CuO/CeO_2_ NCs. Previous reports also suggest that zeta potentials lower than 25 mV would yield a high degree of colloidal stability [50]. Interestingly, the hydrodynamic sizes were much higher than what is expected due to zeta potential. The only rational reason could be the limitations of the DLS analyzer, which can only be sensitive to larger particles. As the TEM result showed, the particles with sizes of nearly 180 nm were observed in the solid phases. Therefore, due to the limitations of the DLS device and the presence of larger particles, it can be concluded that the obtained particle size was plausible. 

### 3.6. Anti-Proliferative Activity of CuO/CeO_2_ NCs against Breast Cancer Cell Lines

**Figure 6:**
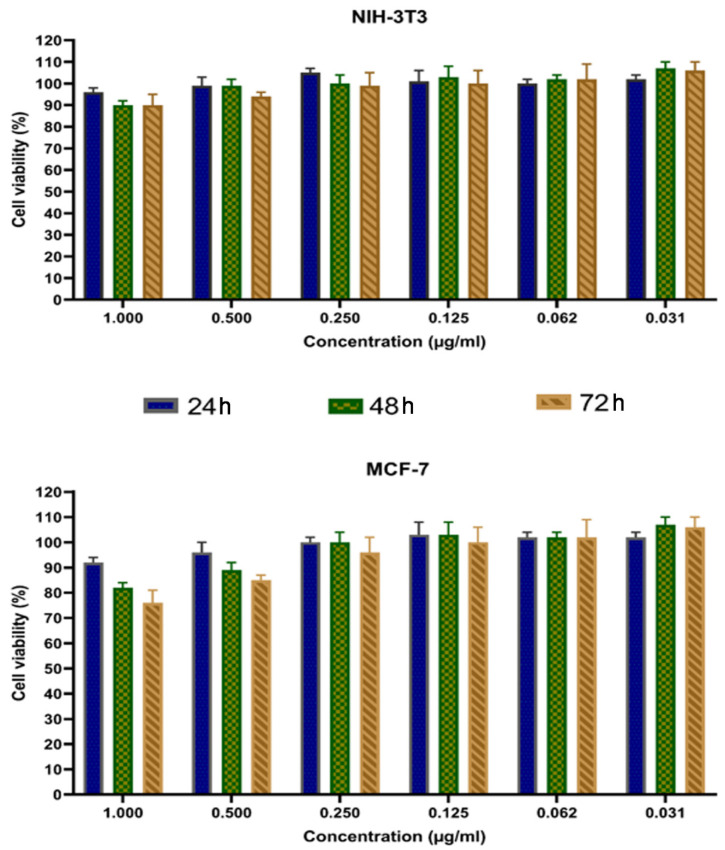
Cytotoxic activity of synthesized CuO/CeO_2_ NCs against normal cell lines (NIH-3T3), and human breast cancer cell line (MCF-7). The percentages are shown as relative to control cells.

Cancer is a leading cause of death and a worldwide health problem [51]. Cancer is a disease characterized by uncontrolled cell proliferation that spreads from an initial focal point to other parts of the body to cause death [52,53]. In the past few decades, the application of nanocomposites in cell toxicity and anticancer properties has attracted a lot of attention with several nanoparticle types being used [54]. In this study, the cell toxicity effect of synthesized CeO_2_-CuO-NPs was measured through MTT assay against a breast cancer cell line (MCF-7) and normal fibroblastic cells. The result of the cytotoxic effect of synthesized nanoparticles (0.031–1.000 μg/mL) is shown in Figure 6 after 24, 48, and 72 h incubation. The results showed that there was a gradual decrease in cell vitality with increasing NC concentration, and this effect was more pronounced for longer incubation times using higher NC concentrations. This trend is clearly visible in cancer cells, where there is a marked decrease of cell viability over time if the NC concentration exceeds 0.250 μg/mL). Overall, the destruction of cancer cells occurs in a state dependent on both concentrations of CuO/CeO_2_ NC and time of exposure. On the contrary, normal fibroblastic cells seem to be less sensitive to the presence of NC. These results are in accordance with the findings reported in some previous studies that are briefly described below.

Es-haghi et al. studied the cell toxicity of CeO NPs and expression of antioxidant genes against liver cancerous cell lines [55]. They found that the biosynthesized cerium nanoparticles had little effect on normal cells (HUVEC) while being able to significantly kill cancer cells. The expression of catalase and superoxide desmutase genes was also increased in this regard. In another research the cytotoxicity of biosynthesized CeO_2_ NPs against MCF-7 breast cancer cell line was investigated. The results of this study revealed the significant inhibitory effects of CeO_2_ NPs on the growth of MCF-7 cells which depended on the concentration and time of treatment [56]. Ahmed et al. studied the effect of CuFe_2_O_4_ NPs against MCF-7 cells. The results showed that the generation of ROS by NPs is generally considered to a major contributor to NPs toxicity [57].

The metabolic features of cancerous cells are not the same as normal cells and, consequently, this can lead to different results in cellular toxicity between normal and cancer cells [3]. Today it is realized that the higher permeation retaining, reduced lymphatic drainage and vasculature leaking of cancerous cells enable the accumulation injected NPs in the tumor tissue [58]. In vitro studies confirmed that CuO NPs cause apoptosis in tumor cells [59]. One of the mechanisms by which CuO NPs destroy cancer cells is the production of reactive hydroxyl ions, which damage the DNA of cancer cells [60]. Another mechanism performed by CuO involves inhibition of Nuclear Factor (NF)-kB (NF-kB). NF-kB has been displayed to have a role in cancer, and so inhibition of this factor plays a significant function in the inhibition of cancer [61].

## 4. Conclusions

CuO/CeO_2_ NCs were successfully synthesized and analyzed from physical and biological viewpoints. It appears the metal–organic framework-based materials as precursors for the synthesis of the metal oxide is a useful and facile method, especially in the synthesis of the mixed metal oxides. The nature of composition and crystalline phases were confirmed by EDX and PXRD analyses. The mean size of composite nanoparticles was 49.18 and 30.58 nm as assessed by FESEM and TEM, respectively. The synthesized CuO/CeO_2_ NC showed cell toxicity properties towards breast cancerous cell lines (MCF-7) in a dose- and time-dependence manner, while the toxicity of CuO/CeO_2_ NC was significantly lower on normal fibroblastic cells.

## Data Availability

Data are included within this article.
